# The Impact of COVID-19 Pandemic Restrictions on Musculoskeletal Pathology Services

**DOI:** 10.7759/cureus.39493

**Published:** 2023-05-25

**Authors:** Ayşe Nur Toksöz Yıldırım, Tulay Zenginkinet, Erhan Okay, Aykut Celik, Zeynep Cagla Tarcan, Muhammed Fevzi Esen, Tolga Onay, Yalçın Turhan, Korhan Özkan, Muhlik Akyurek

**Affiliations:** 1 Pathology, Göztepe Research and Training Hospital, Istanbul, TUR; 2 Pathology, Istanbul Medeniyet University Goztepe Prof. Dr. Suleyman Yalcin City Hospital, Istanbul, TUR; 3 Orthopaedics, Istanbul Medeniyet University Goztepe Prof. Dr. Suleyman Yalcin City Hospital, Istanbul, TUR; 4 Health Information Systems, Institution of Hamidiye Medical Sciences, University of Health Sciences Turkey, Istanbul, TUR; 5 Orthopaedic Surgery and Traumatology, Dr. Lütfi Kırdar Kartal Training and Research Hospital, İstanbul, TUR; 6 Orthopaedics and Traumatology, Duzce University Medical Faculty, Duzce, TUR; 7 Orthopaedics and Traumatology, Medeniyet University Göztepe Training and Reseach Hospital, Istanbul, TUR; 8 Orthopaedics, Maria-Josef-Hospital Greven, Klinik für Orthopadie, Unfall-und Handchirurgie Deutschland, Greven, DEU

**Keywords:** soft tissue lesions, benign bone tumors, bone and soft tissue lesions, trucut biopsy, muskuloskeletal tumors, covid pandemics

## Abstract

Background and objectives: The COVID-19 pandemic has had a negative impact on healthcare in musculoskeletal pathology. There is no standard protocol for pathology services during a pandemic. The study aimed to assess the impact of COVID-19 restrictions on the workload of the musculoskeletal pathology service and the hurdles faced in collaboration with the orthopedic oncology unit in a tertiary reference center in a developing country.

Materials and methods: The pathology reports from mid-March to mid-June 2019, 2020, and 2021 were retrospectively reviewed.

Results: Significant differences were found between the pandemic period (2020) and the non-pandemic periods (2019-2021) in benign bone and soft tissue lesions, resection surgeries, and soft tissue tumors, which were more prevalent in the non-pandemic periods. However, there was no significant decrease in biopsy procedures.

Conclusion*:* During the pandemic period, the biopsy procedure appears to be feasible for bone and soft tissue lesions without the need for anesthesia.

## Introduction

The musculoskeletal oncology service involves a multidisciplinary approach that involves several specialists: musculoskeletal pathologists, orthopedic oncologists, medical-radiation oncologists, and radiologists. It focuses on diagnosing and treating primary benign and malignant soft tissue and bone tumors, as well as metastatic disease of soft tissue and bone [1,2].

The pandemic caused by the coronavirus known as SARS-CoV-2 (severe acute respiratory syndrome coronavirus 2) has affected the entire world, resulting in loss of life, morbidity, and economic decline. Due to limited infrastructure and unpreparedness, many healthcare services and workers have faced numerous problems directly or indirectly related to the COVID-19 (coronavirus disease 2019) pandemic [3,4].

In Turkey, restrictions on social movements were gradually increased starting in mid-March after the first case was reported on March 11, 2020. These restrictions included strict travel bans, quarantine requirements, lockdowns of public places, and even curfews, especially on weekends. In this study, we aimed to assess the impact of COVID-19 restrictions on the workload of the musculoskeletal pathology service and the challenges faced in collaboration with the orthopedic oncology unit at a tertiary reference center in a developing country.

## Materials and methods

The musculoskeletal oncology and pathology service at our hospital is one of the busiest centers in the Asian part of Istanbul.

In this study, reports of all specimens at our hospital's pathology laboratory from mid-March to mid-June 2019, 2020, and 2021 were retrieved from our health information system and classified as biopsy or resection material and either benign or malignant as a primary soft tissue or bone lesion. Metastatic lesions were not included in the study.

All data were entered into Excel sheets (Redmond, USA). The data for 2020 during the lockdown were compared with the equivalent data for the corresponding season in the preceding year (2019), when there was no COVID-19 pandemic, and the following year (2021), when COVID-19 vaccines were introduced, and patients and institutions were starting to adjust to the new reality of the COVID-19 pandemic. No phone call was used to evaluate patients, as the patients with suspected tumors had routine admissions during the pandemic period. 

IBM Corp. Released 2015. IBM SPSS Statistics for Windows, Version 23.0. Armonk, NY: IBM Corp. was used for statistical analysis. The data are given as percentages (%). The Kruskal-Wallis test was performed to compare patient groups according to year, and the two groups were compared using the Mann-Whitney U test. A p-value less than 0.05 was considered significant.

## Results

Between 2019 and 2021, a retrospective review identified 325 cases of bone and soft tissue tumors. The details are shown in Tables 1-3. Two hundred and thirty patients had benign lesions, while 95 had malignant lesions. Biopsy procedures were performed on 30, 32, and 43 patients in 2019, 2020, and 2021, respectively. Resection was performed on 87, 55, and 72 patients in 2019, 2020, and 2021 (Table 1). The localizations included the lower extremity (n=220), upper extremity (n=89), and trunk (n=16), respectively (Table 2). One hundred and seventy-seven patients had bone lesions, while 147 had soft tissue lesions (Table 3). 

**Table 1:** Information regarding intervention and final pathology according to years

Intervention	2019	2020	2021	Total number
Biopsy (benign)	20	19	26	65
Biopsy (malign)	10	13	22	45
Resection (benign)	75	36	53	164
Resection (malign)	12	19	19	50
Malign lesions	22	32	41	95
Benign lesions	95	55	79	229

**Table 2:** Information regarding localization according to the final pathology

	Benign	Malign	Total
Lower extremity	158	62	220
Upper extremity	65	24	89
Trunk	7	9	16

**Table 3:** Final pathology of bone and soft tissue lesions according to the year

	Bone tissue malign (n=64)	Bone tissue benign (n=113)	Soft tissue malign (n=31)	Soft tissue benign (n=117)
2019	15	41	7	54
2020	22	33	10	23
2021	27	39	14	40

There was a statistically significant difference between the pandemic period (2020) and the non-pandemic periods (2019-2021) in resection surgeries (p=0.001) and soft tissue tumors (p=0.028), which were statistically less frequent in the pandemic period (p<0.05). Biopsy procedures did not decrease in the pandemic period (p=0.235, p>0.05) (n=30, 2019; n=33, 2020; n=48, 2021). There was no statistically significant difference in bone biopsy (p=0.833) and bone resections (p=0.052) during the pandemic period (2020) compared to the non-pandemic periods (2019-2021). There was a significant decrease in the frequency of soft tissue tumors in the pandemic period (2020) compared to the non-pandemic period (2019-2021) (p=0.028). There was a steady, significant increase in malignant bone and soft tissue lesions (n=22, 2019; n=32, 2020; n=41, 2021) (malignant bone, p=0.003; malignant soft tissue, p=0.047). There was a statistically significant decrease in all benign bone and soft tissue lesions in the pandemic period compared to the non-pandemic period (n=95, 2019; n=56, 2020; n=79, 2021) (p=0.001, p<0.05). 

## Discussion

The SARS-CoV-2 pandemic has impacted the world and significantly affected the healthcare system and economy. Disruptions have inevitably occurred in the diagnosis and treatment of cancer patients worldwide due to this pandemic. Health authorities around the globe have attempted to keep cancer-related services available during the pandemic, but the reorganization had to be adapted as quickly as possible to keep up with the demanding challenges.

Our hospital is a tertiary reference center that provides healthcare services on the Asian side of Istanbul. During the COVID-19 pandemic, our hospital was selected as one of the care centers for COVID-19, and all elective surgical procedures and interventions were gradually canceled between March 15 and June 15, 2019. However, all emergent surgeries, including oncological cases, were performed. As a multidisciplinary team, radiologists, pathologists, orthopedic oncologists, and medical oncologists remained in the hospital and worked together to minimize disruptions in the care of those patients. In our university hospital, continuous oncologic care with diagnosis and treatment was provided simultaneously with COVID-19 treatment and care. To reduce the risk of exposure of our pathology laboratory staff to COVID-19 infection, a system of working every other day was implemented during working days without causing significant problems, considering the decreased burden of overall pathology specimens as elective cases were postponed or reduced in number. Our two musculoskeletal pathologists, experts in their field, also worked on a rotation basis to assess pathology specimens, order immunohistochemical staining, or perform genetic analyses on frozen sections taken during surgery. Musculoskeletal pathology specimens can sometimes be challenging to diagnose, even for experienced pathologists. In such cases, consultation and mutual discussion with a knowledgeable professional can help overcome diagnostic difficulties. Our musculoskeletal pathology service actively used Zoom cloud meetings to share ideas and consult with an experienced external pathologist. This consultation enabled our team to provide pathology results on time, without any delays that could compromise patients' treatment strategies during the COVID-19 restrictions.

During the most active period of the COVID-19 pandemic, at least one outpatient clinic remained open to care for orthopedic patients who had undergone surgery or conservative treatment after trauma. As a result, patients with possible tumor lesions or newly recognized mass lesions could easily find a point of contact to be referred to our musculoskeletal oncology unit. To minimize outpatient clinic visits and reduce the risk of COVID-19 infections, the cell phone numbers of the senior author or his team members were provided to patients who had undergone surgery or a biopsy. Patients' relatives were encouraged to contact and discuss their pathology results via messaging apps such as WhatsApp or Telegram, which are widely used in the population.

There are only a few published manuscripts in the English-speaking literature that discuss the effect of restrictions during the COVID-19 pandemic on pathology laboratories, specifically regarding the number of specimens dealt with during pandemic lockdowns. To date, there has been no published data on the effect of the COVID-19 pandemic lockdown on the number of specimens in the musculoskeletal oncology pathology service.

Thaler et al. surveyed 149 orthopedic oncology surgeons and found that nearly 20% of all malignant cases were canceled or delayed [1]. This feedback suggests that patient care for sarcoma cases has become dangerous. There was a significant decrease in hospital admissions and surgical interventions in pandemic periods compared to pre-pandemic periods, which is consistent with our results. Alorjani et al. conducted a retrospective study that compared pre-pandemic and pandemic periods [2]. They found a significant reduction (57.9%) in pathologic specimens. They recommended using online pathology platforms based on remote working conditions. The pandemic period will increase the need for developing additional educational resources. Bhat et al. evaluated the effect of the pandemic on dermatology practice [3]. They underlined that effective measures should decrease patient contact by increasing telemedicine use and improving specific clinical guidelines. Prajapati et al. observed a significant decrease in admissions and time to clinical decisions during the pandemic period compared to the non-pandemic period [4]. They found that telemedicine was a safe way to provide patient care in the pandemic group. Gaston et al. retrospectively evaluated cases of osteosarcoma that were present at their institution [5]. They found that there was an increased time to admission and that admissions occurred at later stages of the disease. To mitigate the negative consequences of this, they strongly advised increasing the use of telemedicine, which can connect referral hospitals and local healthcare providers. Fitzgerald et al. found that the three major problems were increased pain, disability, and a delay in diagnosis [6]. They stated that measures should be taken to weigh the risk of COVID-19 infection in hospitals against the risk of uncertainty in treatment. This risk can be minimized by using a reasonable approach. They recommended evaluation based on four levels of the risk scale. Moreover, the pandemic has implemented telemedicine approaches that are frequently used even after pandemics, which helps to decrease costs [7]. During the pandemic, home reporting has become a part of pathology services. The Royal College of Pathologists proposed guidelines to identify and prioritize urgent cases to speed up the home reporting process [8]. Hanna et al. showed a high level of consistency between digital and traditional glass slide pathological assessments. This approach has the potential to improve multi-center collaborations and prepare for future pandemics [9]. The implementation of digital pathology infrastructure began in 2006 at Memorial Sloan Kettering Cancer Center. This system provided flexible and remote work options, which became crucial during the SARS-CoV-2 public health emergency. It has become a foundation for future innovative healthcare delivery models [10]. Additionally, Lujan et al. surveyed pathologists' attitudes toward remote evaluation. Eighteen out of 21 pathologists reported positive feedback for remote primary diagnoses after the pandemic [11]. Despite all these studies, diagnostic procedures such as biopsies remain unchanged. Hospital admissions of the patients are necessary to obtain diagnostic tissue. In the future, home procedures for biopsy could be considered in high-risk elderly cases during pandemics with the necessary equipment for emergent situations.

Our findings and results from the pathology service during the pandemic were compared to those from before the lockdown period in a similar season in 2019. In 2019, there were no pandemics, and healthcare services were not running at full capacity. The results were also compared to a similar season in 2021 when there was still a COVID-19 pandemic, but life was starting to return to normal despite the presence of many infected people, and vaccination had been introduced. As in our study, there was an increased admission rate of malignant cases in the pandemic period (n=32) compared to the pre-pandemic period (n=22) that presented to our hospital despite the pandemic periods. This underlines the need for a remote preassessment of this patient group. Additionally, this group of patients deserves fast evaluation outside of the pandemic period. These practices and the reconstitution of the infrastructure should be revisited in times when there are no pandemics to ensure the care of musculoskeletal oncology patients and the correct diagnosis of their histopathologic specimens. Outside of the pandemic period, telemedicine approaches have been able to decrease patient visits, helping to prevent the acquisition of respiratory diseases in immunocompromised oncology patients. This has caused a permanent change in orthopedic oncology visits. If appropriate measures are taken, emergent surgeries for oncology patients can be performed, followed by the use of telemedicine in pathology services.

Limitations of this study include its retrospective design, small sample size, and lack of comparison based on different tumor subtypes. Tumors can also have an indolent course before they are detected. This study includes data from only one institution. Multicenter data on a national level would be more robust and allow for stronger conclusions to be drawn.

## Conclusions

This study demonstrates the changes in routine pathology services during the pandemic period in terms of the management of bone and soft tissue lesions. During a pandemic, biopsy procedures can be performed in an outpatient setting without the need for anesthesia. This practice can accurately identify malignant cases that require urgent medical care. In future pandemics, it will be important to use resources and the workforce efficiently by improving infrastructure in musculoskeletal pathology and orthopedic oncology services in a tertiary reference center hospital for other emergent conditions.
